# miRNA Sequencing and Differential Analysis of Testis in 1-Year-Old and 2-Year-Old Kazakh Horses

**DOI:** 10.3390/biology15090715

**Published:** 2026-04-30

**Authors:** Yuhe Zhou, Liuxiang Wen, Wanlu Ren, Mingyue Wen, Mengling Ming, Jianwen Wang, Jun Meng, Xinkui Yao, Yaqi Zeng

**Affiliations:** 1 College of Animal Scienc, Xinjiang Agricultural University, Urumqi 830052, China; 15160990694@163.com (Y.Z.); 13890451520@163.com (L.W.); renwanlu@xjau.edu.cn (W.R.); 18280584310@163.com (M.W.); 17308132872@163.com (M.M.); dkwjw@xjau.edu.cn (J.W.); mengjun@xjau.edu.cn (J.M.); yaoxinkui@xjau.edu.cn (X.Y.); 2 Xinjiang Key Laboratory of Equine Breeding and Exercise Physiology, Urumqi 830052, China

**Keywords:** Kazakh horse, testis, transcriptome, DEmiRNAs

## Abstract

The *Kazakh horse* represents a significant genetic resource within China’s *equine* population, characterized by notable resilience and an ability to thrive on coarse forage. Nevertheless, a decline in its numbers has been observed recently, making the improvement of its reproductive performance crucial for the preservation of this breed and the advancement of the related industry. In this study, testicular tissues from 1-year-old (pre-pubertal) and 2-year-old (post-pubertal) *Kazakh horses* were analyzed. miRNA sequencing was conducted on tissues from these age groups, followed by bioinformatics analyses to elucidate the functions of differentially expressed miRNAs (DEmiRNAs). The reliability of the sequencing data was subsequently verified using RT-qPCR. Analysis revealed 165 differentially expressed miRNAs (DEmiRNAs) in the testicular tissues between the two age groups. Of these, 118 DEmiRNAs (e.g., *eca-miR-206* and *eca-miR-2483*) were significantly up-regulated (*p* < 0.05), and 47 DEmiRNAs (e.g., *eca-miR-196a* and *eca-miR-211*) were significantly down-regulated (*p* < 0.05). These DEmiRNAs were mainly implicated in biological processes including lipid metabolism and signal transduction. Their predicted target genes are potentially involved in key reproductive processes, notably testicular development and spermatogenesis. This study identifies candidate miRNAs and potential regulatory pathways associated with sexual maturation in *Kazakh horses*, providing a preliminary molecular basis for future functional validation and improvement of *equine* reproductive performance.

## Simple Summary

Reproductive efficiency is a key factor in the conservation and utilization of *Kazakh horses*, yet the molecular mechanisms underlying testicular development in this breed remain unclear. In this study, we compared the testicular miRNA profiles of pre-pubertal (1-year-old) and post-pubertal (2-year-old) *Kazakh horses*. A total of 165 differentially expressed miRNAs were identified, many of which are potentially involved in pathways related to lipid metabolism, signal transduction, and spermatogenesis. These findings provide novel insights into miRNA-mediated regulatory networks during *equine* sexual maturation and offer candidate molecular markers for improving reproductive performance in *Kazakh horses*.

## 1. Introduction

As a significant indigenous breed in China, the *Kazakh horse* has a substantial population and a broad geographical range [1]. It exhibits traits including consistent genetic traits, high adaptability, and the ability to thrive on roughage [2]. Presently, the breed is extensively utilized for milk and meat production, among other purposes [3]. Enhancing the reproductive efficiency of this valuable indigenous breed is vital to advance its conservation and utilization, which in turn underpins the sustainable development of *equine* genetic resources.

miRNAs are endogenous small RNAs, typically 20–24 nucleotides long, which serve diverse and crucial regulatory functions in cells [4]. Individual miRNAs can have multiple target genes. For instance, research by Guo et al. demonstrated that *miR-181* directly inhibits *Porcine* Reproductive and Respiratory Syndrome Virus (PRRSV) infection in vitro via specific binding to *ORF4* and additionally downregulates expression of the PRRSV receptor *CD163* in blood monocytes and *porcine* alveolar macrophages [5]. Conversely, the expression of a single gene can be modulated by multiple miRNAs. For example, Lai et al. reported that miRNAs including *miR-21*, *miR-146a*, *miR-155*, *miR-222*, and *miR-383* collectively target genes in the NF-κB pathway, thereby synergistically regulating the level of inflammation [6].

Numerous studies to date have characterized the miRNA expression profiles in testicular tissues across various developmental stages in multiple species, including *cattle* [7], *sheep* [8], and *pigs* [9]. By examining *yak* testicular tissues from three critical stages—6 months (juvenile), 18 months (pubertal), and 4 years (adult)—Hu et al. [10] defined stage-specific miRNA expression patterns. Their study identified 1536 miRNAs in total, which included 558 previously known in *yaks*, 717 evolutionarily conserved miRNAs, and 261 novel predicted miRNAs. A comparison of *porcine* testicular tissues at 60 and 180 days of age by Luo et al. [11] revealed 129 differentially expressed miRNAs. For seven of these miRNAs, the expression levels of their predicted target genes involved in spermatogenesis were negatively correlated. Through small RNA deep sequencing of testicular tissues from Southdown × Hu sheep F1 hybrids at 0, 3, 6 months, and 1 year old, Xi et al. [12] identified differentially expressed miRNAs. The target genes of these miRNAs, including *YAP1*, *ITGB1*, and *DOT1L*, were significantly enriched in several signaling pathways crucial for reproduction—such as FOXO, Hippo, Wnt, cAMP, and MAPK—implicating them in key processes like testis development, spermatogenesis, and cellular proliferation and differentiation.

However, systematic studies on miRNA-mediated regulatory networks during testicular development and sexual maturation in *equines* remain very limited. Most available studies focus on adult *horses* or non-reproductive traits, and there is a lack of comprehensive profiling and functional analysis of miRNAs during the critical transition from pre- to post-puberty in *horses*. This knowledge gap restricts the understanding of *equine* reproductive development and the genetic improvement of reproductive efficiency. In *horses*, sexual maturity is typically reached around 2 years of age. Therefore, 1-year-old individuals represent a pre-pubertal stage characterized by incomplete spermatogenesis, whereas 2-year-old individuals exhibit active spermatogenesis and functional reproductive capacity [13]. To fill this key research gap, this study utilized miRNA sequencing to analyze testicular tissues from *Kazakh horses* at pre-pubertal (1 year) and post-pubertal (2 years) stages. We identified differentially expressed miRNAs (DEmiRNAs), predicted their target genes, and performed functional enrichment analysis. The objective was to uncover key regulatory mechanisms underlying testicular development, thereby offering scientific insights to inform future breeding, reproductive management, and conservation strategies for equids.

## 2. Materials and Methods

### 2.1. Sample Collection

Eight *Kazakh horses* reared under uniform management conditions in Tacheng, Xinjiang, were utilized in this study. The *horses* were allocated into two groups based on age: a pre-pubertal group (Group G-1, *n* = 4; aged 1 year) and a post-pubertal group (Group G-2, *n* = 4; aged 2 years). This classification was based on established knowledge that *horses* typically reach sexual maturity at approximately 2 years of age. However, no direct histological or endocrine validation of pubertal status was performed in this study. Tissue samples from the left testis of each animal were surgically excised and promptly preserved in liquid nitrogen for further analysis. All animals were maintained in separate stalls of a common barn, fed with high-quality alfalfa hay and corn grain ad libitum, and allowed free access to clean drinking water.

### 2.2. miRNA Sequencing

Small RNA library construction was then performed. Small RNA fragments (18–30 nt) were first isolated from the total RNA by size selection on a PAGE gel. These fragments underwent sequential ligation of 3′ and 5′ adapters(Illumina-compatible, NEB, USA). The adapter-ligated RNA was reverse-transcribed into double-stranded cDNA, which was then amplified by PCR with specific primers under the following conditions: 94 °C for 30 s; 12 cycles of 94 °C for 15 s, 62 °C for 30 s, 70 °C for 15 s; and a final extension at 70 °C for 5 min. The PCR amplicons were size-selected again on a PAGE gel to purify fragments of approximately 140 bp, which were eluted into EB buffer(10 mM Tris-HCl, pH 8.5). The quality and concentration of the final libraries were evaluated using an Agilent 2100 Bioanalyzer (Agilent Technologies, Santa Clara, CA, USA) and an ABI StepOnePlus Real-Time PCR System (Applied Biosystems, Foster City, CA, USA). Libraries passing quality control were used for sequencing. Library construction and sequencing were conducted by Guangzhou Gidio Biotechnology Co., Ltd. (Guangzhou, China).

### 2.3. Data Quality Control

To reduce the interference from low-quality data, raw reads were processed using the fastp software (v0.23.2) [14,15]. The filtering criteria for read removal were as follows: (1) reads containing adapter sequences; (2) reads in which ambiguous bases (N) constituted more than 10% of the sequence; (3) reads containing polyA sequences were identified and removed to avoid potential bias from polyadenylated fragments; and (4) low-quality reads where more than 50% of the bases had a Phred quality score (Q) ≤20. After filtering, small RNA reads within the expected size range were retained. The remaining reads passing all filtering steps were defined as high-quality reads. Subsequently, redundant sequences were collapsed, and unique sequences were retained as clean tags for downstream analyses.

### 2.4. Analysis of Inter-Sample Correlations

Based on miRNA expression data, Principal Component Analysis (PCA) was conducted utilizing R software (v4.3.1)). This dimensionality reduction technique was used to examine the distances between samples, thereby evaluating the divergence in expression profiles between Groups G-1 and G-2 and the intra-group homogeneity. Correlation analysis was performed to assess the relationships in miRNA expression across samples. The calculated correlation coefficients were then visualized to illustrate the pairwise correlations between samples.

### 2.5. Analysis of DEmiRNAs

DEmiRNAs were identified using the edgeR package (v3.42.4) in R. miRNAs with an absolute log2 fold change (|log2FC|) ≥ 1 and a *p*-value < 0.05 were considered significantly differentially expressed. No multiple testing correction was applied at this stage.

### 2.6. Functional Enrichment Analysis of DEmiRNAs

Enrichment analysis of KEGG pathways was conducted employing the KOBAS (v3.0) tool. The clusterProfiler package (v4.8.1) in R(v4.3.1) was utilized for the graphical representation of significantly enriched GO terms and KEGG pathways. A *p*-value of <0.05 was defined as the cutoff for statistical significance, and *p*-values were adjusted using the false discovery rate (FDR), with FDR <0.05 considered significant.

### 2.7. Target Gene Prediction

The target genes of differentially expressed miRNAs (DEmiRNAs) were predicted using two widely used algorithms: Miranda (v3.3a) and TargetScan (v7.0). For Miranda, the parameters were set as follows: a score threshold ≥140, energy threshold ≤−10 kcal/mol, strict 5′ seed pairing, gap-open penalty of −4.0, and gap-extend penalty of −9.0. TargetScan predicted target genes based on sequence complementarity between the 2–8 nt seed region at the 5′ end of miRNAs and the 3′-UTR of target transcripts. To improve prediction reliability, only the intersection of the results obtained from both methods was retained as high-confidence candidate target genes.

### 2.8. RT-qPCR Validation

For validation of the selected miRNAs, total RNA was reverse-transcribed to generate cDNA. Details regarding the primers used for RT-qPCR are available (see Supplementary Table S1). Quantitative RT-qPCR was conducted using a CFX Connect Real-Time PCR System (Bio-Rad, Hercules, CA, USA), with triplicate measurements for each sample. The relative expression levels of miRNAs were normalized to *U6* small nuclear RNA (*U6*) as an internal reference and calculated using the 2^−ΔΔCt^ method.

## 3. Results and Analysis

### 3.1. miRNA Sequencing Data of Kazakh Horse Testicular Tissue

A total of eight small RNA libraries were constructed and subjected to quality control analysis. As shown in Table 1, the number of clean reads for each sample ranged from 10,985,906 to 15,724,179. Among these, the proportion of high-quality reads ranged from 96.60% to 97.87%, indicating overall high sequencing quality. The proportion of polyA reads was extremely low (0.0008–0.0022%), suggesting minimal contamination by polyadenylated sequences. After further filtering and processing, the proportion of clean tags ranged from 83.42% to 87.99%, which were used for subsequent analyses.

### 3.2. Correlation Analysis Among Testicular Tissue Samples of Kazakh Horses

**Figure 1:**
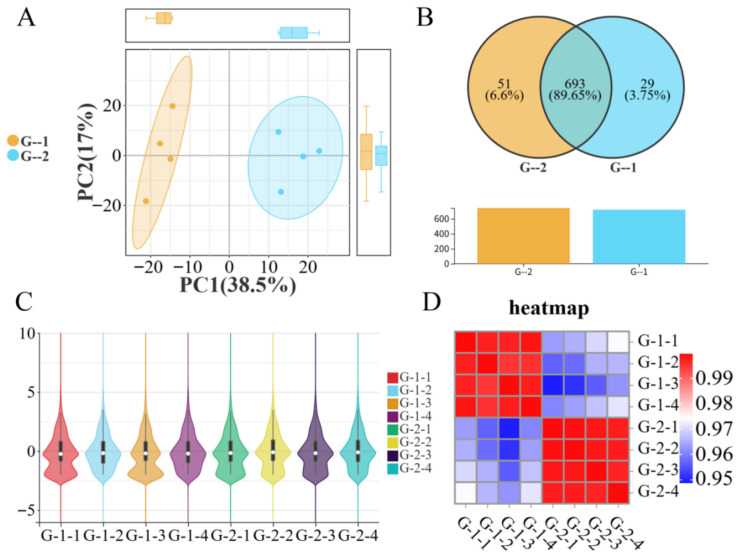
Inter-sample correlation analysis between Group G-1 vs. Group G-2. (**A**) PCA scatter plot between Group G-1 vs. Group G-2 samples; (**B**) Venn diagram between Group G-1 vs. Group G-2; (**C**) violin plot between Group G-1 vs. Group G-2 samples; (**D**) correlation heatmap between Group G-1 vs. Group G-2.

Results from the correlation analysis of testicular tissues from 1- and 2-year-old *Kazakh horses* are shown in Figure 1. Principal component analysis (PCA) revealed that PC1 (the first principal component) explained 38.5% of the variance, and PC2 (the second principal component) explained 17% (Figure 1A). The tight intra-group clustering and clear separation between Group G-1 and Group G-2 indicate distinct expression profiles between the two age groups. Figure 1B shows that 722 and 744 miRNAs were detected in Group G-1 and Group G-2, respectively. Among these, 693 miRNAs, such as *eca-miR-127*, *eca-miR-363*, and *eca-miR-379*, were common to both groups (see Supplementary Table S4). The results presented in Figure 1C,D demonstrate high pairwise correlations among samples within each group, reflecting minimal individual variation and overall consistency in expression levels. A similarly stable correlation trend was observed between the groups.

### 3.3. Differential Expression Analysis in Testicular Tissues of Kazakh Horses

**Figure 2:**
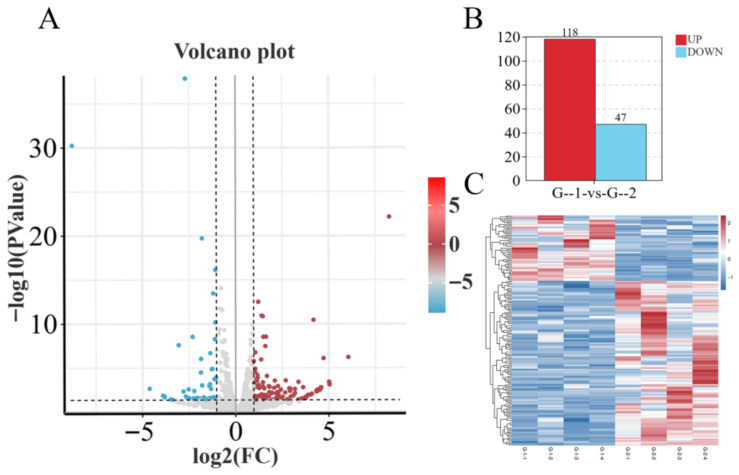
Differential Expression Analysis of the G-1 vs. G-2 Groups. (**A**) Volcano plot of differentially expressed miRNAs between Group G-1 vs. Group G-2. (**B**) Bar chart showing the number of differentially expressed miRNAs between Group G-1 vs. Group G-2. (**C**) Heatmap of differentially expressed miRNAs between Group G-1 vs. Group G-2.

**Figure 3:**
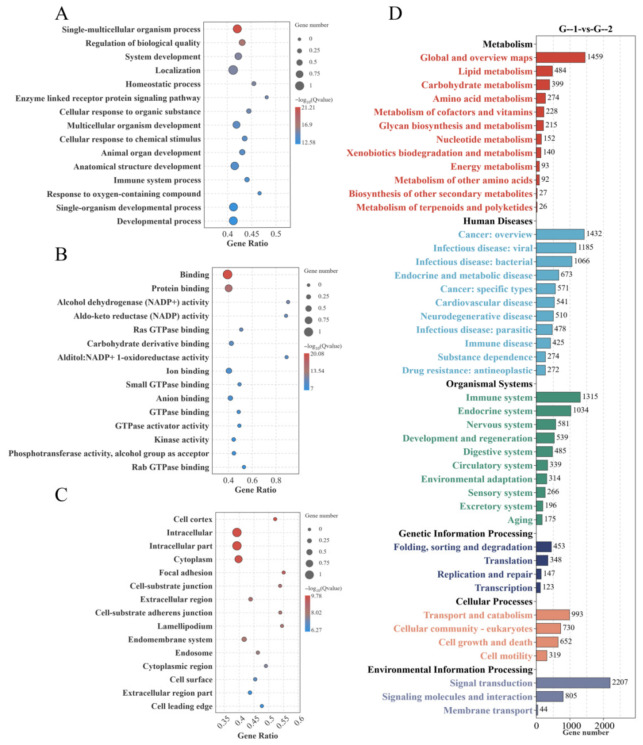
GO and KEGG enrichment analysis for the G-1 vs. G-2 groups. (**A**) Diagram of GO annotation entries for Biological Process (BP) between Group G-1 vs. Group G-2. (**B**) Diagram of GO annotation entries for Molecular Function (MF) between Group G-1 vs. Group G-2. (**C**) Diagram of GO annotation entries for Cellular Component (CC) between Group G-1 vs. Group G-2. (**D**) KEGG enrichment analysis diagram between Group G-1 vs. Group G-2.

Differential expression analysis using the edgeR package (v3.42.4) revealed 165 significant DEmiRNAs, such as *eca-miR-1248*, *eca-miR-1264*, and *eca-miR-1912*, between Groups G-1 and G-2 (Figure 2A,B). Of these, 118 DEmiRNAs (e.g., *eca-miR-206* and *eca-miR-2483*) were up-regulated, and 47 DEmiRNAs (e.g., *eca-miR-196a* and *eca-miR-211*) were down-regulated (*p* < 0.05). Cluster analysis demonstrated high reproducibility among biological replicates and clear distinctions between Group G-1 and Group G-2 (Figure 3C) (see Supplementary Table S2).

### 3.4. Results of GO Annotation and KEGG Enrichment Analysis for Differentially Expressed miRNAs in Kazakh Horse Testicular Tissue

GO annotation of the DEmiRNAs in Groups G-1 and G-2 revealed that these miRNAs were predominantly enriched in several functional categories (Figure 3A–C). These included biological processes (BP) such as single-multicellular organism process and regulation of biological quality, cellular components (CC) like intracellular part and cytoplasm, and molecular functions (MF) encompassing binding and protein binding. KEGG pathway enrichment analysis demonstrated that the DEmiRNAs were significantly associated with pathways including lipid metabolism and signal transduction (Figure 3D) (see Supplementary Table S3).

### 3.5. Prediction Results of miRNA Target Genes

Based on the above target gene prediction method, a total of 85,262 miRNA-target gene pairs were obtained, covering 165 DEmiRNAs and 6336 unique target genes corresponding to 15,154 target transcripts, and the average number of target genes regulated by each miRNA was 516.7; the average binding free energy of all predicted pairs was −22.14 kcal/mol with a median of −21.30 kcal/mol, among which 52,448 pairs accounting for 61.5% of the total had binding free energy ≤−20 kcal/mol, and the average binding score was 0.919 with a median of 0.837, which proved that the predicted results had high stability and reliability. Combined with the same differential significance criteria of DEmiRNAs as used in this manuscript, 2017 high-confidence miRNA–target gene pairs were finally screened out, providing a solid data support for the subsequent exploration of the regulatory role of miRNAs in testicular development and maturation of *Kazakh horses* (see Supplementary Table S5).

### 3.6. Validation Using RT-qPCR

**Figure 4:**
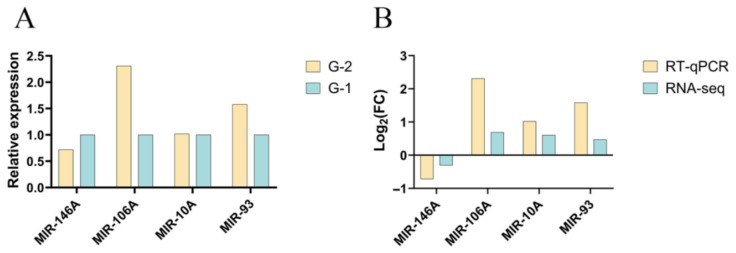
RT-qPCR validation. (**A**) Differential miRNA expression in Group G-1 vs. Group G-2 detected by RT-qPCR. (**B**) Comparison of Log2 fold change (Log2FC) of DEmiRNAs measured by RNA-seq and RT-qPCR.

To validate the accuracy of the miRNA sequencing results, four DEmiRNAs were randomly selected for verification via RT-qPCR. As shown in Figure 4, *miR-146A* was down-regulated, whereas *miR-106A*, *miR-10A*, and *miR-93* were up-regulated. The expression trends of all tested miRNAs were consistent between the RT-qPCR and RNA-seq analyses, confirming the reliability of the sequencing data and supporting their use for further investigation.

## 4. Discussion

Testicular development and spermatogenesis are core processes of male *equine* sexual maturation, which are precisely regulated by post-transcriptional factors such as miRNAs. Our study identified 165 DEmiRNAs between pre-pubertal and post-pubertal *Kazakh horse* testes, suggesting that miRNA-mediated post-transcriptional regulation may be involved in testicular functional maturation. Liu et al. [16] revealed the mRNA expression characteristics of *Mongolian horse* testes at different sexual maturity stages by single-cell RNA sequencing and found that cell structure-related genes were dominant in immature testes, while spermatogenesis-related genes were highly active in mature testes. our results show a generally consistent trend: the target genes of DEmiRNAs screened in this study were significantly enriched in pathways related to cell proliferation, differentiation and reproductive function, indicating that miRNAs and mRNAs form a synergistic regulatory network to drive *equine* testicular maturation. Different from the mRNA-level transcription analysis, this study supplements the miRNA regulatory profile during *equine* testicular development, which fills the research gap of miRNA regulation in *horse* puberty and provides a new molecular perspective for revealing the mechanism of *equine* reproductive development.

*miR-6537-y* and its target gene *APOE* were identified from the high-confidence miRNA-target gene pairs predicted in this study, and they were selected for further discussion due to their potential regulatory roles in testicular development. *APOE*, a predicted target gene of *miR-6537-y*, is a multifunctional protein with expression detected in multiple tissues, among them the testis [17]. Schleicher et al. [18] localized *APOE* within *mouse* testes via immunohistochemistry, revealing prominent expression in Leydig cells and a diffuse distribution in the seminiferous tubules. Their experiments demonstrated that Leydig cells are capable of synthesizing and secreting *APOE*, suggesting a role in local sterol transport. In the process of testosterone biosynthesis, *APOE* may act as a sterol transporter within the testis, facilitating the delivery of cholesterol for hormone synthesis [19]. Additionally, *APOE* has been reported to contribute to maintaining cholesterol metabolic homeostasis and testicular structural integrity [20]. Studies in *APOE*-deficient *mice* further suggest that loss of *APOE* function may impair testicular development and spermatogenesis, as indicated by reduced testosterone levels, altered vascular structure, and decreased sperm production [21,22]. In this study, *miR-6537-y* was significantly up-regulated in post-pubertal testes, suggesting a potential inhibitory effect on its predicted target gene *APOE*. This regulatory relationship may reflect a dynamic adjustment of cholesterol metabolism and testosterone synthesis during testicular maturation. Compared with previous studies that mainly focused on *APOE* protein function, our findings provide preliminary evidence at the miRNA-mediated post-transcriptional regulatory level.

In our study, *MAPK1* was identified as a predicted target gene of several DEmiRNAs, including *eca-miR-196a*, *miR-345-x*, and *miR-735-y*, suggesting that miRNA-mediated regulation may influence MAPK signaling during testicular maturation. *MAPK1* is a known component of the MAPK signaling pathway, which is involved in cell proliferation, differentiation, and spermatogenesis [23,24,25,26,27]. Research by Yamashita et al. [28] involving *mice* with Leydig cell-specific ablation of *MEK1* and *MEK2* revealed a decrease in Leydig cell numbers and reduced expression of key Leydig cell markers, along with diminished testosterone levels, indicating the importance of MAPK signaling in testicular function. In addition, MAPK signaling has been reported to regulate the proliferation and differentiation of spermatogonial cells in multiple species [29,30,31]. In this study, the identified miRNAs showed significant expression differences between the two age groups, suggesting that they may regulate the expression of *MAPK1* and may be related to differences in testicular maturation between one-year-old and two-year-old *Kazakh horses*. It should be noted that the target gene is only bioinformatically predicted and lacks functional experimental validation, and the above speculation requires further verification. These findings are generally consistent with previous studies demonstrating the importance of MAPK signaling in spermatogenesis. However, our study suggests that this pathway may be regulated at the miRNA level in *equine* testes.

The equilibrium between spermatogonial proliferation and apoptosis is important for maintaining normal spermatogenesis, and its disruption can impair testicular function [32]. *STK4*, a predicted target of miRNAs including *eca-miR-350*, *miR-4301-z*, and *miR-452-x* is a pro-apoptotic kinase involved in regulating apoptotic signaling pathways [33,34]. Previous studies have shown that disruption of *STK4* function can impair apoptosis regulation and affect spermatogenesis. For example, mutations in *STK4* have been reported to disrupt apoptotic processes [35], and *STK4* deficiency in animal models has been associated with infertility, abnormal sperm development, and dysregulated cell proliferation and apoptosis [36]. In this study, the differential expression of miRNAs targeting *STK4* suggests that miRNA-mediated regulation may contribute to maintaining the balance between germ cell proliferation and apoptosis during testicular maturation in *Kazakh horses*. However, these regulatory relationships are based on bioinformatic prediction and require further experimental validation.

Although our findings are generally consistent with previous transcriptomic studies, some differences in specific miRNA expression patterns and regulatory relationships were observed. These discrepancies may be attributed to species-specific genetic backgrounds, differences in developmental stage selection, and methodological variations such as bulk RNA sequencing versus single-cell RNA sequencing. These factors may collectively contribute to the observed diversity in regulatory mechanisms.

While this study provides insights into miRNA expression during testicular development, several limitations should be acknowledged. First, the classification of animals into pre-pubertal and post-pubertal groups was based solely on age, without direct histological or endocrine validation of testicular maturity. Although this approach is consistent with established knowledge of *equine* sexual development, it may not fully capture individual variation in pubertal timing. Second, the relatively small sample size (*n* = 4 per group) limits the statistical power and does not support definitive mechanistic conclusions. Third, the analysis is largely correlative, and the regulatory relationships between miRNAs and their predicted target genes require further functional validation.

## 5. Conclusions

miRNA sequencing analysis of pre- and post-pubertal *Kazakh horse* testes identified 165 differentially expressed miRNAs, including key up-regulated miRNAs (e.g., *eca-miR-206* and *eca-miR-2483*) and down-regulated miRNAs (e.g., *eca-miR-196a* and *eca-miR-211*). Target gene prediction suggested that these miRNAs may regulate genes such as *APOE*, *MAPK1*, and *STK4*, which are involved in lipid metabolism, signal transduction, and spermatogenesis. These findings suggest that miRNA-mediated regulatory networks may be associated with the transition from pre-pubertal to post-pubertal stages in *Kazakh horses*. However, these regulatory relationships remain putative and require further experimental validation. Overall, this study provides a basis for understanding *equine* testicular development and offers candidate targets for future functional and breeding-related studies.

## Figures and Tables

**Table 1:** Overall detection of miRNA sequencing data.

Sample	Clean_Reads	High_Quality	polyA	Clean_Tags
G-1-1	10,185,392 (100%)	9,909,222 (97.2886%)	84 (0.0008%)	8,918,393 (87.5606%)
G-1-2	15,724,179 (100%)	15,204,,732 (96.6965%)	217 (0.0014%)	13,250,115 (84.2659%)
G-1-3	11,966,079 (100%)	11,559,130 (96.5991%)	172 (0.0015%)	10,337,437 (86.3895%)
G-1-4	14,691,054 (100%)	14,377,864 (97.8682%)	159 (0.0011%)	12,926,605 (87.9896%)
G-2-1	11,497,853 (100%)	11,106,655 (96.5976%)	174 (0.0016%)	9,876,716 (85.9005%)
G-2-2	10,989,506 (100%)	10,624,537 (96.6789%)	233 (0.0022%)	9,218,960 (83.8888%)
G-2-3	11,606,989 (100%)	11,219,529 (96.6618%)	186 (0.0017%)	9,684,736 (83.4388%)
G-2-4	13,226,692 (100%)	12,814,437 (96.8832%)	230 (0.0018%)	11,033,589 (83.4191%)

## Data Availability

The data presented in this study are openly available in BioProject (reference number: PRJNA1377007).

## Supplementary Materials

The following supporting information can be downloaded at: https://www.mdpi.com/article/10.3390/biology15090715/s1, Table S1: Primers used for RT-qPCR validation; Table S2: Differentially expressed miRNAs; Table S3: GO and KEGG enrichment analysis results; Table S4: Shared and unique miRNAs between groups; Table S5: High-confidence miRNA–target gene pairs.

## References

[1] Ren W., Wang J., Zeng Y., Wang T., Meng J., Yao X. (2024). Differential age-related transcriptomic analysis of ovarian granulosa cells in Kazakh horses. Front. Endocrinol..

[2] Yu X., Fang C., Liu L., Zhao X., Liu W., Cao H., Lv S. (2021). Transcriptome study underling difference of milk yield during peak lactation of Kazakh horse. J. Equine Vet. Sci..

[3] Ren W., Wang J., Zeng Y., Wang T., Sun Z., Meng J., Yao X. (2024). Investigating age-related differences in muscles of Kazakh horse through transcriptome analysis. Gene.

[4] Gan M., Liu L., Zhang S., Guo Z., Tan Y., Luo J., Yang Q., Pan H., Li X., Wang J. (2021). Expression characteristics of microRNA in pig umbilical venous blood and umbilical arterial blood. Animals.

[5] Guo X.-k., Zhang Q., Gao L., Li N., Chen X.-x., Feng W.-h. (2013). Increasing expression of microRNA 181 inhibits porcine reproductive and respiratory syndrome virus replication and has implications for controlling virus infection. J. Virol..

[6] Lai Y.-C., Fujikawa T., Maemura T., Ando T., Kitahara G., Endo Y., Yamato O., Koiwa M., Kubota C., Miura N. (2017). Inflammation-related microRNA expression level in the bovine milk is affected by mastitis. PLoS ONE.

[7] Gao Y., Li S., Lai Z., Zhou Z., Wu F., Huang Y., Lan X., Lei C., Chen H., Dang R. (2019). Analysis of long non-coding RNA and mRNA expression profiling in immature and mature bovine (*Bos taurus*) testes. Front. Genet..

[8] Wu J., Zhu H., Song W., Li M., Liu C., Li N., Tang F., Mu H., Liao M., Li X. (2014). Identification of conservative microRNAs in Saanen dairy goat testis through deep sequencing. Reprod. Domest. Anim..

[9] Zhang B., Yan Z., Gao Y., Li J., Wang Z., Wang P., Yang Q., Huang X., Gun S. (2022). Integrated analysis of miRNA and mRNA expression profiles in testes of Landrace and Hezuo boars. Front. Vet. Sci..

[10] Hu L., Wang X., Guo S., Cao M., Kang Y., Ding Z., Pei J., Ge Q., Ma Y., Guo X. (2024). Whole-transcriptome sequencing analysis to identify key circRNAs, miRNAs, and mRNAs in the development of yak testes. BMC Genom..

[11] Luo L., Ye L., Liu G., Shao G., Zheng R., Ren Z., Zuo B., Xu D., Lei M., Jiang S. (2010). Microarray-based approach identifies differentially expressed microRNAs in porcine sexually immature and mature testes. PLoS ONE.

[12] Xi B., An X., Yue Y., Shen H., Han G., Yang Y., Zhao S. (2025). Identification and profiling of microRNAs during sheep’s testicular development. Front. Vet. Sci..

[13] Naden J., Amann R.P., Squires E.L. (1990). Testicular growth, hormone concentrations, seminal characteristics and sexual behaviour in stallions. Reproduction.

[14] Chen S., Zhou Y., Chen Y., Gu J. (2018). fastp: An ultra-fast all-in-one FASTQ preprocessor. Bioinformatics.

[15] Lu Z., Wen M., Yao X., Meng J., Wang J., Zeng Y., Li L., Ren W. (2025). Differential analysis of testicular LncRNA in Kazakh horses of different ages. Int. J. Biol. Macromol..

[16] Liu Y., Du M., Li X., Zhang L., Zhao B., Wang N., Dugarjaviin M. (2024). Single-cell transcriptome sequencing reveals molecular expression differences and marker genes in testes during the sexual maturation of mongolian horses. Animals.

[17] Tréguier Y., Bull-Maurer A., Roingeard P. (2022). Apolipoprotein E, a crucial cellular protein in the lifecycle of hepatitis viruses. Int. J. Mol. Sci..

[18] Schleicher R.L., Zheng M., Zhang M. (1993). Immunocytochemical localization and endogenous synthesis of apolipoprotein E in testicular Leydig cells. Biol. Reprod..

[19] Shatwan I.M., Winther K.H., Ellahi B., Elwood P., Ben-Shlomo Y., Givens I., Rayman M.P., Lovegrove J.A., Vimaleswaran K.S. (2018). Association of apolipoprotein E gene polymorphisms with blood lipids and their interaction with dietary factors. Lipids Health Dis..

[20] Ravenhill S.M., Evans A.H., Crewther S.G. (2023). Escalating bi-directional feedback loops between proinflammatory microglia and mitochondria in ageing and post-diagnosis of Parkinson’s disease. Antioxidants.

[21] Steinfeld K., Beyer D., Mühlfeld C., Mietens A., Eichner G., Altinkilic B., Kampschulte M., Jiang Q., Krombach G.A., Linn T. (2018). Low testosterone in ApoE/LDL receptor double-knockout mice is associated with rarefied testicular capillaries together with fewer and smaller Leydig cells. Sci. Rep..

[22] Langheinrich A.C., Paradowska A., Kilinski R., Kampschulte M., Steinfeld K., Altinkilic B., Steger K., Stieger P., Bergmann M., Weidner W. (2012). Mixed testicular atrophy related to atherosclerosis: First lessons from the ApoE^−/−^/LDL receptor^−/−^ double knockout mouse model. Int. J. Androl..

[23] Tian J., Xue B., Hu J., Li J., Cheng X., Hu J., Li F., Chen Y., Li B. (2017). Exogenous substances regulate silkworm fat body protein synthesis through MAPK and PI3K/Akt signaling pathways. Chemosphere.

[24] Yue J., López J.M. (2020). Understanding MAPK signaling pathways in apoptosis. Int. J. Mol. Sci..

[25] Jing J., Ding N., Wang D., Ge X., Ma J., Ma R., Huang X., Jueraitetibaike K., Liang K., Wang S. (2020). Oxidized-LDL inhibits testosterone biosynthesis by affecting mitochondrial function and the p38 MAPK/COX-2 signaling pathway in Leydig cells. Cell Death Dis..

[26] Zhang H., Liu Y., Xu K., Mao K., Han W., Xu F., Wan W., Sun Y. (2019). AMPH-1 as a critical tumor suppressor that inhibits osteosarcoma progression. Cancer Manag. Res..

[27] Ishaq Y., Ikram A., Alzahrani B., Khurshid S. (2022). The Role of miRNAs, circRNAs and Their Interactions in Development and Progression of Hepatocellular Carcinoma: An *Insilico* Approach. Genes.

[28] Yamashita S., Tai P., Charron J., Ko C., Ascoli M. (2011). The Leydig cell MEK/ERK pathway is critical for maintaining a functional population of adult Leydig cells and for fertility. Mol. Endocrinol..

[29] Meng X., Lindahl M., Hyvönen M.E., Parvinen M., de Rooij D.G., Hess M.W., Raatikainen-Ahokas A., Sainio K., Rauvala H., Lakso M. (2000). Regulation of cell fate decision of undifferentiated spermatogonia by GDNF. Science.

[30] Morimoto H., Iwata K., Ogonuki N., Inoue K., Atsuo O., Kanatsu-Shinohara M., Morimoto T., Yabe-Nishimura C., Shinohara T. (2013). ROS are required for mouse spermatogonial stem cell self-renewal. Cell Stem Cell.

[31] Sahare M., Otomo A., Komatsu K., Minami N., Yamada M., Imai H. (2015). The role of signaling pathways on proliferation and self-renewal of cultured bovine primitive germ cells. Reprod. Med. Biol..

[32] Wang E.H., Yu Z.L., Bu Y.J., Xu P.W., Xi J.Y., Liang H.Y. (2019). Grape seed proanthocyanidin extract alleviates high-fat diet induced testicular toxicity in rats. RSC Adv..

[33] Shimazaki M., Wittayarat M., Sambuu R., Sugita A., Kawaguchi M., Hirata M., Tanihara F., Takagi M., Taniguchi M., Otoi T. (2022). Disruption of cell proliferation and apoptosis balance in the testes of crossbred cattle-yaks affects spermatogenic cell fate and sterility. Reprod. Domest. Anim..

[34] Lin C., Hsu T., Chiou P., Hsiao M., Wang W., Chen Y., Lin J., Wang J., Lin P., Lin F. (2020). Downregulation of STK4 promotes colon cancer invasion/migration through blocking β-catenin degradation. Mol. Oncol..

[35] Abolnezhadian F., Iranparast S., Ahmadpour F. (2020). Identical twins with a mutation in the STK4 gene showing clinical manifestations of the mutation at different ages: A case report. Iran. J. Immunol..

[36] Meng C., Tian G., Xu C., Li X., Zhang Y., Wang Y., Qin J., Fok E.K.L., Hinton B.T., Mak K.K.-L. (2020). Hippo kinases MST1 and MST2 control the differentiation of the epididymal initial segment via the MEK-ERK pathway. Cell Death Differ..

